# No Weekend Effect in Elective Primary Total Knee Arthroplasty: A Nationwide Analysis of 437,121 U.S. Cases

**DOI:** 10.3390/jcm14248816

**Published:** 2025-12-12

**Authors:** David Maman, Yaniv Steinfeld, Yaron Berkovich

**Affiliations:** 1 Carmel Medical Center, Haifa 3436212, Israel; 2 Faculty of Medicine, Technion Israel Institute of Technology, Haifa 2611001, Israel

**Keywords:** total knee arthroplasty, weekend effect, elective surgery, postoperative complications, hospital readmission, perioperative outcomes, NRD, health services research

## Abstract

**Background**: The “weekend effect” describes the concern that patients treated on weekends experience worse outcomes due to differences in staffing, resource availability, and workflow. Evidence for a weekend effect in elective orthopedic surgery is limited and inconsistent, and most prior work does not ensure that surgery itself actually occurs on the weekend. We aimed to evaluate whether weekend admission and surgery are associated with worse in-hospital or 90-day outcomes in a contemporary nationwide cohort of elective primary total knee arthroplasty performed on hospital day 0. **Methods**: We conducted a retrospective cohort study using the U.S. Nationwide Readmissions Database (NRD) from 2020 to 2022. Adult patients (≥18 years) undergoing elective primary TKA with surgery on hospital day 0 were identified using ICD-10-PCS procedure codes in the primary procedure position. Weekend admissions (Saturday–Sunday) were compared with weekday admissions (Monday–Friday). Baseline demographics, comorbidities, and hospital characteristics were assessed. Outcomes included length of stay, total hospital charges, in-hospital mortality, major postoperative complications, and 90-day all-cause readmissions, time to readmission, readmission length of stay, and procedures during readmission. Continuous variables were compared using *t*-tests and categorical variables using chi-square or Fisher’s exact tests (two-sided α = 0.05). **Results**: Among 437,121 elective day-0 TKA admissions, 435,822 (99.7%) occurred on weekdays and 1299 (0.3%) on weekends. Baseline characteristics were highly similar between groups. No clinically meaningful differences were observed in in-hospital complications, mortality, or 90-day readmission outcomes. Small statistical differences in blood transfusion, blood-loss anemia, and postoperative pain did not follow a pattern consistent with a weekend effect. **Conclusions**: In this large contemporary national cohort of elective primary TKA with surgery on hospital day 0, weekend admission and surgery were not associated with worse in-hospital outcomes or higher 90-day readmission rates. Within standardized perioperative pathways, elective TKA appears safe when performed on weekends, without evidence of a weekend effect.

## 1. Introduction

Total knee arthroplasty (TKA) is one of the most commonly performed and resource-intensive orthopedic procedures, with steadily rising utilization in the United States and worldwide [1,2,3]. As surgical volume continues to grow, optimizing perioperative quality, minimizing preventable complications, and reducing unnecessary readmissions have become central priorities for hospitals and health systems [4,5]. Contemporary care pathways for TKA increasingly emphasize standardized protocols, enhanced recovery after surgery (ERAS) programs, and close coordination between surgeons, anesthesiologists, nursing staff, and rehabilitation teams [3,4,5].

Against this background, the “weekend effect” has emerged as an important organizational concern. The term refers to the possibility that patients admitted or treated on weekends experience inferior outcomes due to reduced staffing, limited access to ancillary services, and altered workflow patterns compared with weekdays [6,7]. Evidence of a weekend-associated risk signal has been reported across several high-acuity populations, including stroke, acute coronary syndromes, and trauma, where weekend admission has been linked to higher mortality or complication rates [8,9,10]. More recent large database and health services studies have continued to explore the weekend effect across a broad range of surgical and medical conditions, with generally mixed findings regarding its magnitude and clinical relevance.

In orthopedics, the weekend effect has been most extensively studied in urgent and emergent settings. Hip fracture and spine trauma series have suggested that weekend presentation or surgery may be associated with higher mortality, complications, or delays to definitive care in some contexts [6,7]. However, elective orthopedic surgery differs fundamentally from emergency trauma care: case selection is planned, perioperative optimization is possible, and protocolized clinical pathways are common. Several studies examining elective lower limb arthroplasty have therefore reported more nuanced results. Some have found no evidence of a weekend effect in elective orthopaedic care [11], whereas others, including recent analyses using administrative datasets, have reported longer length of stay, higher costs, or increased complication rates when TKA or total joint arthroplasty (TJA) is performed on weekends [12]. These conflicting findings underscore the need for contemporary, procedure-specific evaluations.

Methodological limitations may partly explain the heterogeneity of prior arthroplasty studies. First, many series have combined urgent and elective procedures or pooled different joints (hip and knee), potentially obscuring procedure-specific patterns. Second, several investigations have relied on pre-COVID-19 data that may not reflect current ERAS protocols, outpatient pathways, and modern perioperative optimization [3,4,5,11]. Third and perhaps most importantly, nearly all prior work has defined exposure by day of admission rather than day of surgery. Under this design, many patients admitted on a weekend undergo their operation on the subsequent weekday, meaning their surgical care does not occur under true weekend staffing conditions, introducing substantial exposure misclassification.

Under modern elective arthroplasty pathways, patients typically undergo admission and surgery on the same calendar day. Restricting the cohort to hospital-day-0 procedures therefore provides a more accurate and clinically meaningful measure of weekend operative care. Despite the importance of TKA as a high-volume, protocol-driven procedure, no contemporary nationwide study has specifically evaluated whether weekend surgery influences postoperative complications, in-hospital outcomes, or 90-day readmissions in a homogeneous cohort of elective, primary, hospital-day-0 TKA patients in the post-COVID era [11,12].

Accordingly, we used the U.S. Nationwide Readmissions Database (NRD) from 2020 to 2022 to examine the association between weekend admission (and thus weekend surgery) and outcomes after elective primary TKA performed on hospital day 0.

Weekend elective surgery remains uncommon in the United States, largely due to institutional staffing models and scheduling policies that prioritize weekday operative activity.

The present study therefore examined whether weekend admission, and consequently weekend surgery, adversely affects in-hospital outcomes, postoperative complications, or 90-day readmissions among adults undergoing elective primary TKA.

## 2. Methods

### 2.1. Study Design and Data Source

We performed a retrospective cohort study using the Healthcare Cost and Utilization Project (HCUP) Nationwide Readmissions Database (NRD) for the years 2020–2022. The NRD is an encounter-level, all-payer claims database that captures approximately 60% of all U.S. inpatient hospitalizations and enables tracking of patients across hospitals within a calendar year through encrypted linkage identifiers. Each hospitalization is associated with discharge weights that allow generation of nationally representative estimates, along with up to 40 diagnosis fields and 25 procedure fields coded using ICD-10-CM/PCS.

### 2.2. Cohort Identification

We identified adult patients (≥18 years) who underwent primary TKA using ICD-10-PCS procedure codes listed in the primary procedure field (PR1). Elective admissions were selected using the NRD admission type variable. To obtain a clean and homogeneous elective TKA cohort that reflects modern perioperative pathways, we applied the following criteria

Inclusion

- Elective admission
- Primary TKA in PR1
- Surgery performed on hospital day 0 (PRDAY0 = 0)

Exclusion

- Nonelective or trauma admissions
- Revision or bilateral TKA
- Fracture, infection, or neoplasm at the time of admission
- COVID-19 diagnosis (U07.1)
- Patients <18 years old
- Discharges after September (insufficient 90-day follow-up)

A total of 437,121 elective day-0 primary TKA admissions met criteria.

### 2.3. Exposure Definition: Weekend vs. Weekday Admission

The main exposure was day of admission:

- Weekday: Monday–Friday
- Weekend: Saturday–Sunday

This definition aligns with prior “weekend effect” studies and reflects typical U.S. hospital staffing structures. In the NRD, day of admission corresponds to the calendar date of the surgical procedure for hospital-day-0 elective cases, ensuring that no weekday spillover surgeries were misclassified as weekend procedures. Elective TKA is occasionally performed on weekends in selected institutions, though it remains uncommon nationwide. Procedures performed after midnight are coded by calendar day, preventing cross-day misclassification.

### 2.4. Variables and Covariates

From the index hospitalization, we extracted demographic, clinical, and hospital characteristics, including:

- Demographics: age (years), sex, primary expected payer, and patient urban-rural residence category based on National Center for Health Statistics (NCHS) codes.
- Comorbidities: hypertension, dyslipidemia, type 2 diabetes, chronic kidney disease (CKD), chronic lung disease, congestive heart failure (CHF), liver disease, chronic anemia, obesity, osteoporosis, and obstructive sleep apnea. These were identified using ICD-10-CM diagnosis codes in the primary and secondary diagnosis fields (DX1-DXn) based on previously published code lists.
- Full ICD-10 diagnosis and procedure code lists are provided in Supplementary Table S1.
- Hospital characteristics: hospital teaching status and bed size, and U.S. census division, as provided in NRD hospital-level variables.

A detailed description of diagnosis and procedure codes can be provided in supplementary material to facilitate replication.

### 2.5. Outcomes

Measured outcomes included length of stay (LOS), total hospital charges, and postoperative complications-specifically deep vein thrombosis (DVT), pulmonary embolism (PE), acute kidney injury (AKI), sepsis, pneumonia, urinary tract infection (UTI), respiratory failure, intraoperative fracture, surgical site infection, blood loss anemia, postoperative pain (G89.18), and blood transfusion. Ninety-day outcomes included all-cause readmission, days to readmission, readmission LOS and readmission requiring a procedure.

### 2.6. Statistical Analysis

Continuous variables are presented as means with standard deviations (SD) and were compared using independent-samples *t*-tests. Categorical variables are presented as counts with percentages and were compared using chi-square tests; Fisher’s exact test was used when expected cell counts were <5. Statistical significance was defined as a two-sided *p* < 0.05. Analyses were conducted using IBM SPSS Statistics 26 (IBM Corp., Armonk, NY, USA). Where appropriate, discharge-level NRD weights can be applied to generate nationally representative estimates; in this analysis, we focus on observed (unweighted) comparisons to preserve transparency of the absolute event counts.

Baseline characteristics between weekday and weekend cohorts were highly similar, and no clinically meaningful imbalances were observed across demographic, clinical, or hospital variables. Given this intrinsic similarity and the very small size of the weekend cohort, multivariable adjustment or propensity-score methods were unlikely to meaningfully alter effect estimates. Therefore, unadjusted comparisons were considered acceptable as the primary analytic approach.

### 2.7. Ethics Approval and Data Use Compliance

The study used fully de-identified data from the HCUP NRD. Per HCUP and federal policy, research using NRD data does not constitute human subjects research and does not require individual informed consent. All analyses complied with the HCUP Data Use Agreement, and no attempt was made to identify individual patients or institutions. Institutional policies classified this project as non-human-subjects research.

## 3. Results

A total of 437,121 elective day-0 primary TKA admissions met inclusion criteria. Of these, 435,822 (99.7%) occurred on weekdays and 1299 (0.3%) occurred on weekends. Baseline demographic, clinical, and hospital characteristics were highly similar between groups (Table 1). Age and sex distribution were nearly identical, and no clinically meaningful differences were observed across primary payer type, urban-rural location, or any major comorbidity category. Robotic-assisted TKA was used at similar rates in both cohorts.

### 3.1. In-Hospital Outcomes

In-hospital outcomes were comparable between weekday and weekend admissions (Table 2). Length of stay was similar (2.09 vs. 2.22 days), with a small absolute difference that did not represent a clinically meaningful effect. Total hospital charges were also comparable. In-hospital mortality was extremely rare in both groups, with no difference observed. Hospital characteristics such as teaching status, bed size, and geographic region were similar between groups, suggesting that weekend cases were not concentrated within a single hospital type; however, the small weekend cohort limits detailed subgroup analysis.

Postoperative complications including deep vein thrombosis, pulmonary embolism, acute kidney injury, sepsis, pneumonia, urinary tract infection, respiratory failure, intraoperative fracture, and surgical site infection occurred at low rates in both groups, with no significant or clinically meaningful differences. Although statistically significant *p*-values were noted for blood transfusion, blood-loss anemia, and postoperative pain, their absolute differences were small and not consistent with a weekend effect.

### 3.2. Ninety-Day Outcomes

Ninety-day outcomes were also similar between groups (Table 3). All-cause readmission occurred in 5.9% of weekend versus 5.3% of weekday admissions. Time to readmission and length of stay during readmission did not differ significantly. The proportion of readmissions requiring a procedure was nearly identical in both groups.

Across all evaluated inpatient and 90-day outcomes, no clinically meaningful weekend effect was identified in elective primary TKA. The absolute differences in 90-day outcomes were small, including a 0.6% difference in readmission rates, which is not clinically meaningful.

## 4. Discussion

### 4.1. Key Observations

In this large, contemporary national cohort of more than 437,000 elective TKA procedures, weekend admission and surgery were not associated with worse in-hospital outcomes, postoperative complications, or 90-day readmissions. These findings suggest that, under modern perioperative pathways, elective TKA can be performed safely on weekends without evidence of a weekend effect.

Although statistically significant differences were observed for blood-loss anemia and transfusion, the absolute differences were small (approximately 3% for anemia and <1% for transfusion) and below thresholds typically considered clinically meaningful in perioperative TKA studies. The discordant pattern—lower anemia but higher transfusion—may reflect coding variation or differing institutional transfusion thresholds rather than a true weekend effect. Small statistical differences were identified for transfusion, blood-loss anemia, and postoperative pain; the absolute risk differences were modest (e.g., <1% for transfusion). The direction and magnitude of these differences did not follow a pattern consistent with a true weekend effect, and the effect sizes fall well below thresholds generally considered clinically meaningful in perioperative TKA literature.

### 4.2. Comparison with Prior Literature

Previous studies evaluating weekend outcomes in surgical populations have reported mixed findings. Some investigations observed higher mortality or complication rates for weekend admissions in emergency and high-acuity conditions, including stroke, myocardial infarction, and trauma [12]. In orthopedics, earlier studies often combined elective and urgent procedures, covered multiple joints, or predated enhanced recovery and standardized care pathways [13,14]. Many also relied on day of admission rather than day of surgery, introducing potential misclassification, because elective arthroplasty patients admitted on weekends frequently undergo surgery on weekdays [15].

Our study overcomes this limitation by restricting the cohort to hospital-day-0 elective TKA, ensuring that weekend admissions represent weekend surgery. Within this refined definition, we found no evidence that weekend operative care compromises safety, complication risk, or readmission outcomes. These results align with more recent single-center and registry studies reporting comparable perioperative performance between weekend and weekday elective orthopedic procedures [12].

### 4.3. Interpretation and Clinical Implications

The absence of a weekend effect in elective primary TKA likely reflects several contemporary factors: standardized operative workflows, enhanced recovery protocols, multimodal analgesia, improved perioperative nursing coverage, and structured postoperative monitoring. Elective TKA is a highly protocolized operation, and deviations in staffing patterns during weekends may have limited practical impact on care delivery.

For hospitals seeking to expand surgical capacity or optimize scheduling flexibility, these results support the feasibility and safety of performing elective TKA on weekends. However, the extremely low proportion of weekend cases in this dataset suggests that weekend elective surgery remains uncommon nationally and may reflect institutional policies rather than patient selection. These findings suggest that weekend elective surgery may help increase surgical capacity in high-volume systems without compromising short-term safety.

### 4.4. Strengths and Limitations

Strengths of this study include its large national sample, restriction to elective day-0 surgery to avoid exposure misclassification, and evaluation of contemporary post-COVID-19 datasets that reflect current perioperative practice. The NRD structure also enabled robust assessment of 90-day readmissions.

Limitations include reliance on administrative coding, which may miss subtle clinical details or misclassify complications. The weekend cohort was small relative to the weekday cohort. Unmeasured confounders such as surgeon experience, staffing patterns, or intraoperative efficiency could not be captured. The NRD does not provide implant details, functional outcomes, or outpatient complications. Finally, the observational design precludes establishing causality. Although the weekend cohort was small, baseline characteristics were nearly identical between groups, reducing the likelihood of meaningful confounding. Prior methodological literature has shown that when exposure groups demonstrate minimal measurable imbalance and event rates are low, adjusted and unadjusted estimates are often comparable [16]. For transparency, we elected to present unadjusted results, consistent with several recent NRD-based arthroplasty studies. Because only 0.3% of elective TKA cases occurred on weekends, these findings may primarily reflect practice patterns at the minority of institutions that routinely perform weekend elective surgery, and thus generalizability may be limited. Formal sensitivity analyses (e.g., exclusion of very short LOS or stratification by hospital characteristics) were considered but were not expected to meaningfully change the results given the extremely low weekend case volume and the near-identical baseline characteristics. Nevertheless, future work using datasets with larger weekend cohorts could incorporate such analyses. Although baseline characteristics were highly similar, small absolute differences in comorbidities such as obesity and diabetes may still influence postoperative risk. Residual and unmeasured confounding, including factors such as staffing patterns, surgeon caseload, and intraoperative workflow, cannot be excluded. Because only 1299 weekend cases were available, the study was underpowered to detect very rare complications such as mortality, pulmonary embolism, or sepsis. ICD-10 coding may misclassify certain subjective outcomes, such as postoperative pain, and the NRD does not capture complications treated exclusively in outpatient or emergency settings, which may reduce sensitivity for events like DVT or PE occurring after discharge.

## 5. Conclusions

In this nationwide cohort of more than 437,000 elective primary TKA procedures performed on hospital day 0, weekend admission and surgery were not associated with higher rates of in-hospital complications, postoperative morbidity, or 90-day readmissions. Outcomes were highly comparable between weekend and weekday cases, indicating that elective TKA can be performed safely on weekends within modern standardized perioperative pathways.

This study has limitations, including reliance on administrative coding, the small weekend cohort, and the inability to capture surgeon-specific or staffing-related variables. Future research should evaluate whether these findings generalize to other elective orthopedic procedures and to institutions with different resource and staffing models.

Overall, our results support the safety and feasibility of performing elective primary TKA on weekends without evidence of a weekend effect.

## Figures and Tables

**Table 1:** Baseline characteristics of weekday vs. weekend admissions.

Characteristic	Weekday (*n* = 435,822)	Weekend (*n* = 1299)	*p*-Value
Age, mean ± SD	67.62 ± 9.54	67.43 ± 9.86	0.46
Female sex	61.2%	60.9%	0.80
Hypertension	57.8%	57.9%	0.92
Diabetes	22.3%	20.2%	0.08
Dyslipidemia	51.5%	51.0%	0.76
CKD	9.3%	8.5%	0.37
CHF	1.4%	1.8%	0.33
Chronic lung disease	6.1%	5.9%	0.69
Chronic anemia	5.8%	4.9%	0.15
Obesity	35.7%	33.1%	0.05
Osteoporosis	4.5%	4.6%	0.81
Obstructive sleep apnea	16.0%	16.2%	0.85
Robotic-assisted TKA	9.9%	11.5%	0.07

**Table 2:** In-hospital outcomes.

Outcome	Weekday	Weekend	*p*-Value
Length of stay (days)	2.09 ± 2.16	2.22 ± 2.55	0.024
Total hospital charges (USD)	71,925	75,154	0.021
In-hospital mortality	0%	0%	0.52
DVT	0.1%	0.2%	0.35
PE	0.2%	0.5%	0.056
AKI	1.9%	2.2%	0.32
Sepsis	0.1%	0.1%	0.35
Pneumonia	0.2%	0.2%	0.12
UTI	0.7%	0.4%	0.21
Respiratory failure	0%	0%	0.77
Intraoperative fracture	0.4%	0.3%	0.76
Surgical site infection	0%	0.1%	0.29
Blood transfusion	1.1%	1.7%	0.047
Blood-loss anemia	11.9%	8.8%	0.001
Postoperative pain (G89.18)	4.0%	5.5%	0.007

**Table 3:** Ninety-day outcomes.

Outcome	Weekday	Weekend	*p*-Value
All-cause readmission	5.3%	5.9%	0.35
Days to readmission	29.8 ± 25.4	34.8 ± 28.3	0.085
Readmission LOS (days)	4.85 ± 5.20	4.70 ± 3.93	0.80
Procedure during readmission	3.2%	3.4%	0.72

## Data Availability

The NRD dataset used in this analysis is publicly accessible from the Healthcare Cost and Utilization Project (HCUP) upon request and data-use agreement.

## Supplementary Materials

The following supporting information can be downloaded at: https://www.mdpi.com/article/10.3390/jcm14248816/s1. Table S1: ICD-10-CM and ICD-10-PCS codes used to define the elective primary TKA study cohort and exclusion criteria.
