# Supplementary material for: No Weekend Effect in Elective Primary Total Knee Arthroplasty: A Nationwide Analysis of 437,121 U.S. Cases
Source: J Clin Med. 2025 Dec 12;14(24):8816. doi: 10.3390/jcm14248816 (PMC12733472; doi:10.3390/jcm14248816)
Supplement: Supplementary file 1 [file jcm-14-08816-s001.zip › jcm-4023613-supplementary.pdf]

Table S1. ICD-10-CM and ICD-10-PCS codes used to define the elective primary TKA study cohort and exclusion criteria.

Category	ICD-10 Code(s)	Description
**Primary TKA (procedure)**	0SRC0J90SRD0J90SRB0J9	Replacement of right/left knee joint with synthetic substitute, cemented, open approach (ICD-10-PCS standard codes for primary TKA)
**Exclude: Fracture**	S72.x, S82.x	Femur or tibia/fibula fractures
**Exclude: Neoplasm**	C40.x, C41.x	Malignant neoplasm of bone and cartilage
**Exclude: COVID-19**	U07.1	SARS-CoV-2 (confirmed COVID-19)
**Exclude: Age < 18**	—	Administrative exclusion using NRD age field
